# Placental Thickness Correlates with Severity-Weighted Fetal Dysfunction in the Third Trimester

**DOI:** 10.3390/jcm14217461

**Published:** 2025-10-22

**Authors:** Julia Murlewska, Oskar Sylwestrzak, Iwona Strzelecka, Łukasz Sokołowski, Paulina Kordjalik, Maciej Słodki, Maria Respondek-Liberska

**Affiliations:** 1 Department of Prenatal Cardiology, Polish Mother’s Memorial Hospital Research Institute, Rzgowska 281/289, 93-338 Lodz, Poland; sylwestrzakoskarpatryk@gmail.com (O.S.);; 2 Department of Obstetrics and Gynecology, Polish Mother’s Memorial Hospital Research Institute, 93-338 Lodz, Poland; 3 Faculty of Public Health, Department for Fetal Malformations Diagnoses & Prevention, Medical University of Lodz, 90-419 Lodz, Poland; 4 Faculty of Health Sciences, The Masovian State University, 09-400 Plock, Poland

**Keywords:** placental thickness, fetal echocardiography, prenatal ultrasound

## Abstract

**Simple Summary:**

This research uncovered that embryos presenting augmented placental thickness, especially those surpassing 70 mm, exhibited heightened incidences of both operational cardiac and extracardiac complications. By attributing weights to each diagnosis in accordance with clinical severity, we established that placental thickness is associated with the cumulative burden of fetal dysfunctions. Although a correlation between thick placentae and structural cardiac anomalies was not strongly established, such placentae were more frequently linked to indicators of fetal distress, including atypical blood flow, regurgitation, or soft tissue abnormalities. Measurement of placental thickness is a straightforward process during standard ultrasound examinations and may function as an early marker of fetal jeopardy, especially during the third trimester.

**Abstract:**

**Background:** Placental thickness has been associated with adverse perinatal outcomes, but the relationship to specific fetal abnormalities seems to not yet be well understood. This study investigates whether increased placental thickness correlates with the severity of fetal cardiac and extracardiac conditions using a structured classification and severity-weighted scoring system. **Methods:** We undertook a retrospective analysis of 1452 fetal echocardiograms conducted during the third trimester at a tertiary referral institution from the years 2022 to 2025. The diagnoses were categorized into four distinct classifications: congenital heart anomalies, cardiac dysfunctions, extracardiac malformations, and extracardiac dysfunctions. Each diagnostic category was allocated a severity weight predicated on established fetal and neonatal mortality risk literature. The evaluation of placental thickness was regarded not merely as a persistent variable but also categorized into three distinct classifications: thin (≤40 mm), intermediate (41–69 mm), and thick (≥70 mm). The examination of correlations was performed utilizing Spearman’s ρ; comparative evaluations among the groups were conducted employing the Kruskal–Wallis and Mann–Whitney U tests. **Results:** Placental thickness revealed a moderate positive correlation with weighted extracardiac dysfunctions (ρ = 0.36, *p* < 0.00001), displayed a comparatively weaker yet statistically significant association with cardiac dysfunctions (ρ = 0.13, *p* = 0.01). Fetuses identified by increased placental thickness (≥70 mm) exhibited notably higher mean scores for both cardiac and extracardiac dysfunctions. Within the cohort exhibiting thick placentas, 25.8% displayed extracardiac dysfunction scores surpassing 0.3, in contrast to only 7.7% within the cohort with thinner placentas. **Conclusions:** Augmented placental thickness correlates with an elevated cumulative load of fetal dysfunction, especially in the realms of extracardiac and functional cardiac impairments. The measurement of placental thickness may function as a straightforward, supplementary indicator of fetal distress in the third trimester, particularly when utilized alongside targeted imaging modalities.

## 1. Introduction

The structure and appearance of the placenta offer insights into the conditions inside the womb, potentially acting as a straightforward, non-invasive way to gauge fetal well-being. When the placenta is unusually sized or shaped, it’s often linked to various complications, such as restricted fetal growth, signs of distress in the baby, and other issues around birth. One notable aspect is an increase in placental thickness, which shows up in pregnancies affected by conditions like diabetes, infections, anemia, fluid buildup, or genetic issues [1,2,3,4,5,6,7,8,9,10,11,12,13,14,15,16]. That said, it’s still not clear how meaningful placental thickness is in situations where no other explanations are apparent.

Fetal echocardiography provides detailed insight into both structural and functional aspects of cardiac development. While congenital heart defects (CHDs) have been extensively studied in relation to genetic and environmental risk factors [17,18,19], the role of the placenta in fetal cardiac physiology has received comparatively less attention. Some studies have proposed that placental dysfunction may precede or parallel fetal cardiac dysfunction, particularly in cases of cardiac hypertrophy, valve insufficiency, or abnormal blood flow [20,21,22,23,24].

Most prior research was focused only on the association of placental thickness with birthweight or pregnancy complications [1,2,3,4,5,6,7,8,9,10,11,12,13,14,15,16]. Few studies have systematically examined its relationship to specific fetal anomalies or dysfunctions, particularly within the context of advanced fetal imaging.

The aim of this study was to evaluate whether increased placental thickness correlates with the presence and severity of fetal abnormalities, including cardiac and extracardiac findings, in a third-trimester population referred for specialized fetal echocardiography. To enhance clinical relevance, we applied a condition classification system and weighted scoring model that prioritizes diagnoses by severity rather than simple frequency. This approach allowed us to test whether placental overgrowth might reflect not just abnormal anatomy but accumulated physiologic stress in the fetus. To our knowledge, this is the first study to combine a structured diagnostic framework with severity-weighted fetal pathology in relation to placental thickness.

## 2. Material and Methods

**Figure 1:**
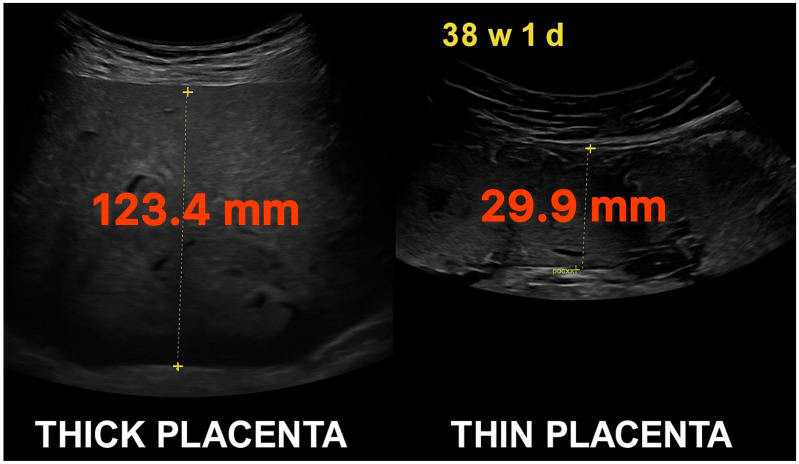
Comparative ultrasonographic images of placental thickness at 38 weeks and 1 day of gestation. Left: markedly thick placenta measuring 123.4 mm. Right: thin placenta measuring 29.9 mm. Both measurements were taken from the anterior uterine wall using standardized perpendicular caliper placement.

This is a retrospective observational study, which was conducted to assess whether placental thickness is associated with fetal cardiac and extracardiac outcomes, using both correlation analysis and group comparisons across defined condition classifications. We analyzed fetal echocardiographic data from the Department of Prenatal Cardiology at the Polish Mother’s Memorial Hospital Research Institute. All included examinations were specialized fetal echocardiograms referred to due to abnormal screening ultrasound findings, maternal comorbidities (e.g., obesity, diabetes, hypertension, infections, or thyroid disease), or family history of congenital heart disease. These were not routine third-trimester obstetric scans but targeted evaluations performed by fetal cardiology specialists. Between 1 January 2022, and 14 March 2025, fetal echocardiograms were performed for 2082 Polish and Ukrainian pregnant patients. Of these, 1452 (52.3%) had recorded placental thickness measurements and were included in the analysis. Placental thickness (PT) was measured on a perpendicular cross-section, from the top to the bottom edge of the placenta, excluding the cord insertion site (Figure 1). Examinations with gestational age below 28 weeks were excluded, as third-trimester measurements are more clinically stable and placental thickness was not routinely documented earlier. Patients were stratified into three PT categories: Thin: ≤40 mm (662 cases; 45.6%), Intermediate: 41–69 mm (679 cases; 46.8%), Thick: ≥70 mm (99 cases; 6.8%). To assess diagnostic outcomes, each patient’s ultrasound report was reviewed for the presence of structural or functional abnormalities. Diagnoses were categorized into four major condition classifications: congenital heart defects (CHD), cardiac dysfunctions (functional impairments such as cardiomegaly, regurgitation, or flow abnormalities), extracardiac malformations (ECMs), and extracardiac dysfunctions (soft findings such as ventriculomegaly, polyhydramnios, or fetal growth restriction). Each individual diagnosis was mapped to exactly one condition classification. Because not all conditions are equally severe, a weighted scoring system was introduced to improve clinical granularity. We assigned weights to each condition based on estimated severity and mortality impact, informed by clinical literature and expert consensus (Table 1, Table 2, Table 3 and Table 4) [25,26,27]. These weights were applied across patients to compute cumulative scores per classification, allowing a more nuanced analysis than simple condition counts. This approach was especially important in detecting patterns where high-severity conditions were disproportionately associated with abnormal placental morphology. Cardiac dysfunction was defined as the presence of at least two of the following: cardiomegaly, myocardial hypertrophy, pericardial effusion, disproportion in ventricular or great vessel size, tricuspid regurgitation, ductal constriction, bidirectional or reversed Doppler flows (e.g., through foramen ovale or aortic isthmus), arrhythmias, or intracardiac calcifications. This threshold was chosen to avoid overclassifying mild or isolated findings. ECMs were defined as structural anomalies outside the heart, including central nervous system (CNS), gastrointestinal, genitourinary, musculoskeletal, and facial/neck defects. This follows typologies established by Chang et al. (2021) [28], who found similar distributions of extracardiac involvement among fetuses with congenital heart disease. Exclusion criteria included multiple pregnancies, fetuses with known chromosomal syndromes (e.g., Down syndrome [n = 8], DiGeorge [n = 1], Patau [n = 1]), cases of antiphospholipid syndrome (n = 1), nicotine use in pregnancy (n = 1), pregestational diabetes with vascular complications, and any placenta located on the posterior or lateral wall, low-lying, previa, or partially abrupted.

## 3. Statistical Analysis

The statistical examination concentrated on discerning relationships between placental thickness and classifications of fetal conditions by employing both continuous correlation techniques and categorical group comparison methodologies. In the principal analysis, placental thickness was regarded as a continuous variable.

Each diagnosis was first mapped to one of four condition classifications (congenital heart defects, cardiac dysfunctions, extracardiac malformations, and extracardiac dysfunctions). Conditions were not treated equally: each was assigned a mortality-based severity weight derived from clinical literature and expert consensus. Weights were assigned to each diagnosis based on relative fetal or neonatal mortality risk, drawn from published epidemiological studies and national outcome data [25,26,27]. Conditions with higher reported fatality rates received proportionally greater weight, allowing the scoring system to reflect clinical severity rather than raw frequency. For each patient, total weighted scores were computed by summing the severity weights of all diagnoses present within each classification. This cumulative scoring approach allowed for more nuanced analysis than simple condition counting.

Correlation between placental thickness and classification-specific weighted scores was assessed using Spearman’s rank correlation coefficient ρ (rho) given the non-normal distribution and ordinal nature of many variables. The results of the correlation analysis were depicted through heatmaps, where the color red represented positive correlations and blue denoted negative or inverse associations. Additionally, scatterplots accompanied by fitted regression lines were employed to demonstrate the nature and magnitude of these associations.

Group comparisons across three placental thickness categories (Thin ≤ 40 mm, Intermediate 41–69 mm, Thick ≥ 70 mm) were conducted using one-way ANOVA or the non-parametric Kruskal–Wallis test, depending on data distribution. When appropriate, post hoc pairwise comparisons were performed using the Mann–Whitney U test. Mean weighted scores and standard deviations were reported for each group, along with the percentage of cases falling within each thickness range. No formal a priori power calculation was performed, as this was a retrospective study including all available cases from the database. Statistical processing and visualization were conducted using Python 3.11 with the pandas, scipy, seaborn, and matplotlib libraries.

## 4. Results

Among the 1452 cases with reported placental thickness, the mean thickness was 42.5 ± 19.3 mm, measured at a mean gestational age of 35.5 ± 3 (39.6-largest and 28-smallest) weeks. Placental thickness was stratified into three categories: Thin (n = 662, 45.6%), Intermediate (n = 679, 46.8%), and Thick (n = 99, 6.8%).

### Weighted Scores Across Placental Thickness Groups

**Chart 1:**
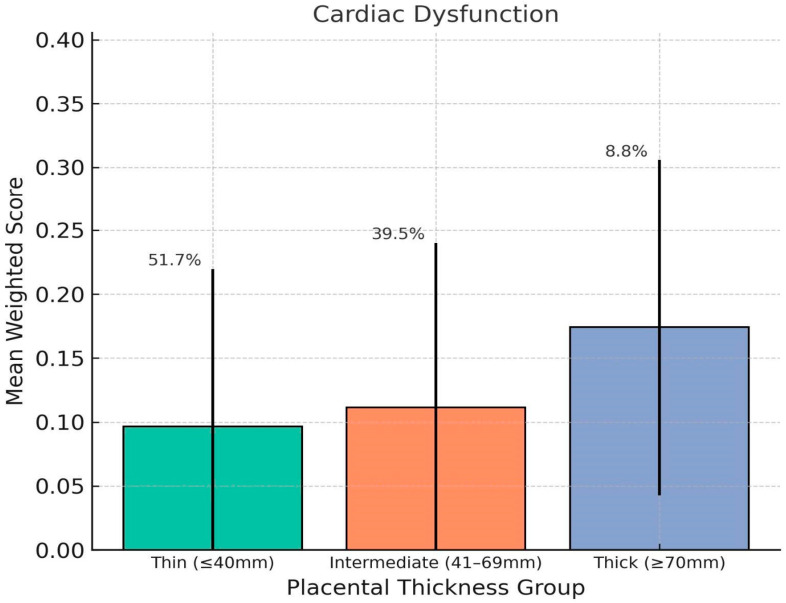
This chart shows how placental thickness relates to the weighted severity of fetal cardiac dysfunction. We’ve plotted the average weighted scores for three groups based on thickness: thin (≤40 mm), intermediate (41–69 mm), and thick (≥70 mm). The error bars show the standard deviation, and the percentages above each bar represent the percentage of fetuses in that category. Overall, fetuses with thicker placentas had the highest average scores for cardiac dysfunction, which points to a greater likelihood of functional heart issues in that group.

**Chart 2:**
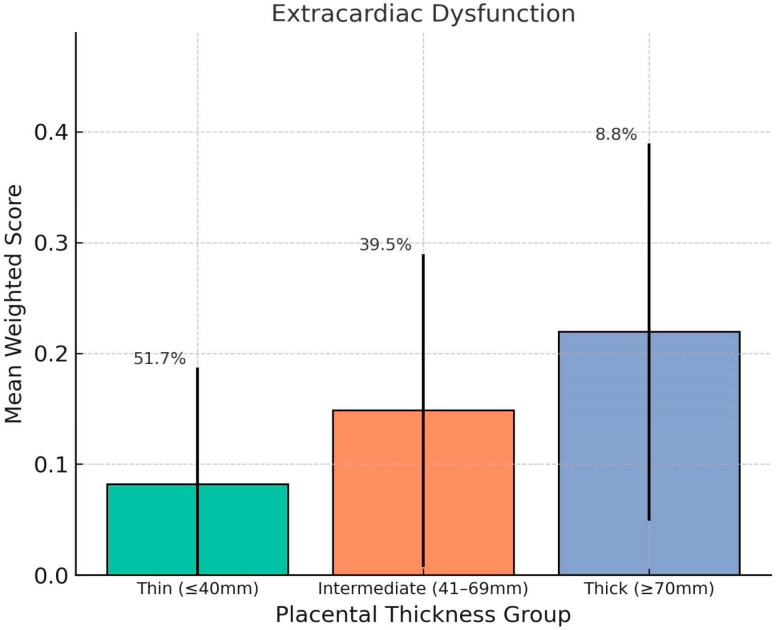
This chart shows the association between placental thickness and weighted severity of fetal extracardiac dysfunction. The mean weighted scores are displayed for the Thin (≤40 mm), Intermediate (41–69 mm), and Thick (≥70 mm) placental thickness groups, with error bars representing standard deviation. The thick placenta group showed the highest average score, indicating a stronger association with systemic fetal dysfunctions such as ventriculomegaly, pyelectasis, and amniotic fluid volume abnormalities. Group percentages denote the proportion of fetuses in each thickness category.

**Chart 3:**
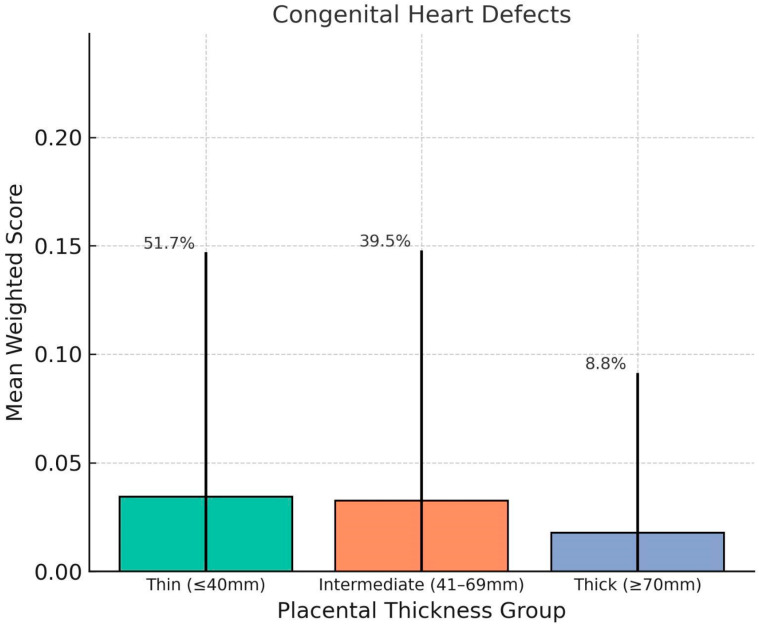
This chart shows comparison of mean weighted scores for Congenital Heart Defects (CHDs) across placental thickness groups. No clear trend was observed, and the thick placenta group exhibited the lowest average CHD score. This finding supports the hypothesis that placental overgrowth is more closely linked with dynamic or functional fetal impairments rather than early-arising structural malformations. Error bars represent standard deviation; percentages indicate group distribution.

**Chart 4:**
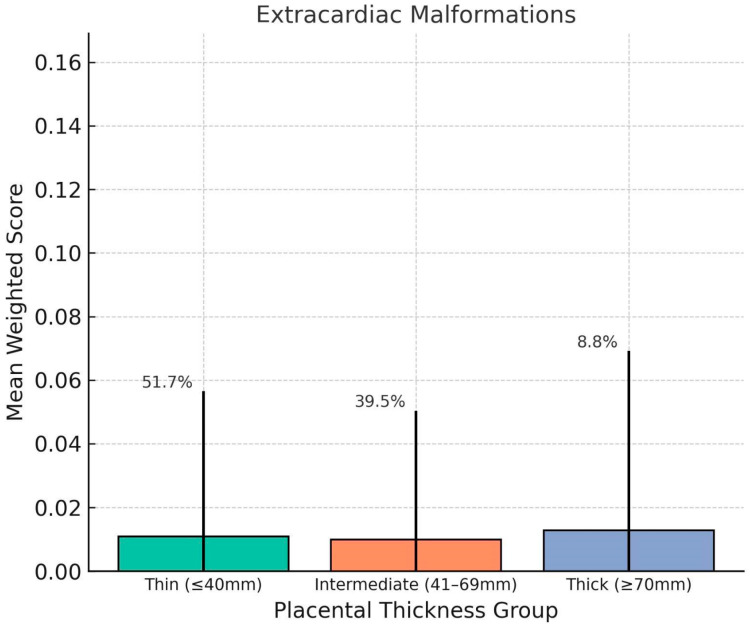
This distribution of mean weighted scores for Extracardiac Malformations (ECMs) across placental thickness groups. No significant differences were observed among the groups, and average scores remained low regardless of placental thickness. This suggests that ECMs, which are structural anomalies of non-cardiac systems, may not be directly influenced by placental morphology in the third trimester. Error bars indicate standard deviation; group percentages denote distribution of the study population.

**Chart 5:**
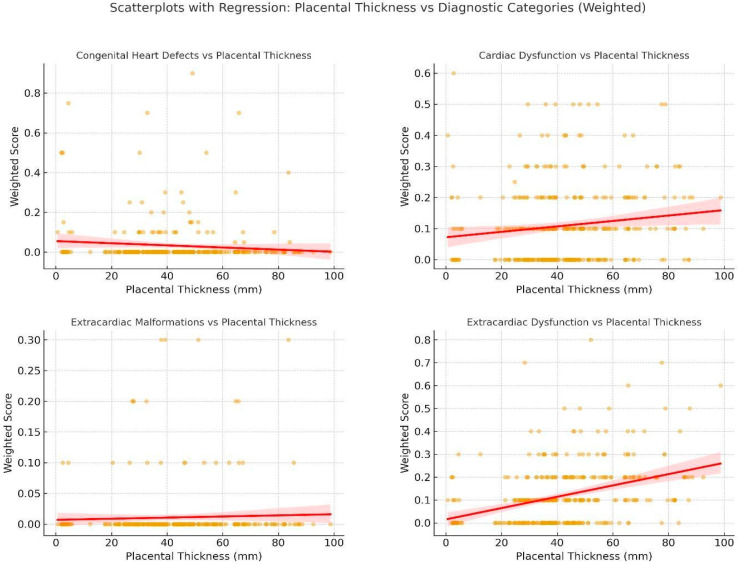
These are scatterplots with regression lines showing the relationship between placental thickness and weighted diagnostic scores for four Condition Classifications: Congenital Heart Defects, Cardiac Dysfunction, Extracardiac Malformations, and Extracardiac Dysfunction. Each dot represents an individual fetus; red lines indicate fitted regression with 95% confidence intervals. Strongest correlations were observed for extracardiac dysfunction (ρ = 0.36) and cardiac dysfunction (ρ = 0.13), while structural anomalies (CHDs, ECMs) showed weak or absent associations with placental thickness.

Mean mortality-weighted scores were calculated for each condition classification within each thickness group. Fetuses with thick placentas (≥70 mm) showed significantly higher weighted scores for cardiac dysfunctions (mean = 0.17, SD = 0.13), extracardiac dysfunctions (mean = 0.30, SD = 0.23). Compared to the Thin group (cardiac dysfunctions = 0.10, extracardiac dysfunctions = 0.14), these differences were statistically significant (*p* < 0.01). Congenital heart defects and extracardiac malformations showed less pronounced variation between groups (Chart 1, Chart 2, Chart 3, Chart 4 and Chart 5).

**Figure 2:**
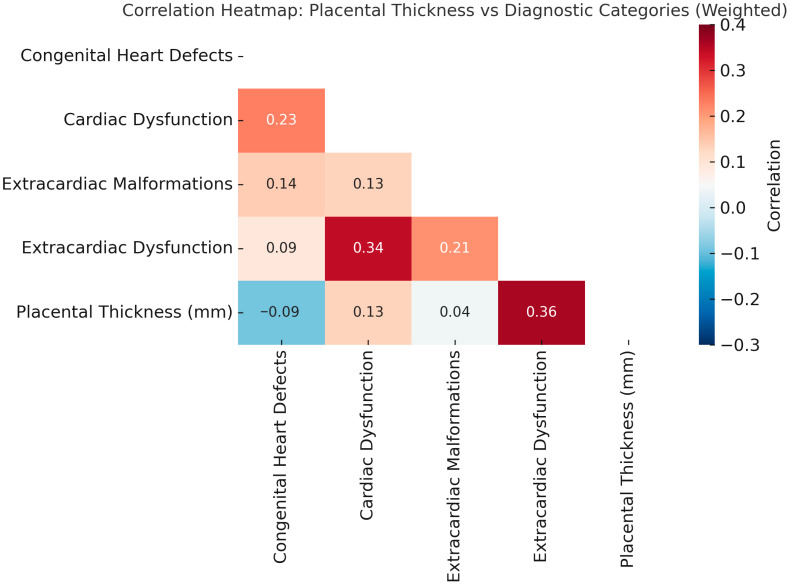
Correlation Heatmap: Placental Thickness vs. Diagnostic Categories (Weighted). Spearman correlation coefficients (ρ) are shown within each cell. The strongest correlation was observed between placental thickness and extracardiac dysfunction (ρ = 0.36), followed by cardiac dysfunction (ρ = 0.13). Structural anomalies, including congenital heart defects and extracardiac malformations, demonstrated weak or absent correlations. Color intensity reflects the strength and direction of the association.

**Correlation Analysis:** Using Spearman’s rank correlation, placental thickness showed the strongest association with extracardiac dysfunctions (rho = 0.36, *p* < 0.00001) and cardiac dysfunctions (rho = 0.13, *p* = 0.01). Scatterplots revealed a moderate upward trend in these classifications, with heavier clustering of high scores among patients with thick placentas. CHD and ECM scores showed no statistically meaningful correlation with placental thickness (Figure 2).

**Distribution of Findings by Group:** Among fetuses with thick placentas, the proportion of cases with high-weight functional abnormalities was notably elevated. The proportion of patients with extracardiac dysfunctions exceeding a weighted score of 0.3 was 25.8% in the thick group, compared to 7.7% in the thin group.

These findings suggest a clinically meaningful association between placental overgrowth and cumulative burden of fetal conditions, particularly for systemic and cardiac soft markers.

## 5. Discussion

### 5.1. Principal Findings

This research delineates a statistically meaningful association between augmented placental thickness and the aggregate severity of both fetal cardiac and extracardiac dysfunctions. Through the implementation of mortality-adjusted weights assigned to distinct diagnoses, we effectively encompassed not merely the existence of anomalies but also their comparative clinical significance. This weighting approach revealed clearer associations than simple condition counting, particularly in the thick placenta group.

The strongest correlation was observed between placental thickness and extracardiac dysfunctions, with a Spearman ρ (rho) of 0.36. A quarter of fetuses in the thick placenta group had weighted extracardiac dysfunction scores exceeding 0.3, more than three times the rate observed in the thin group. This pattern suggests that placental overgrowth may be a marker of underlying systemic fetal stress or dysregulation.

Interestingly, the relationship between placental thickness and structural congenital heart defects (CHD) was weak, while functional cardiac impairments (e.g., regurgitation, cardiomegaly, abnormal flow patterns) were more strongly associated. This distinction aligns with the hypothesis that placental changes may reflect physiologic consequences of fetal dysfunction rather than the presence of static anatomic malformations [20,21,22,23,24].

From a clinical perspective, placental thickness is easily measurable during third-trimester ultrasound and may provide a low-cost, supplementary marker of fetal well-being [29,30,31,32]. While not diagnostic on its own, a thick placenta (particularly >70 mm) should prompt closer scrutiny for signs of fetal compromise, including both cardiac and systemic soft markers.

These findings support further prospective studies to determine whether integrating placental thickness into third-trimester surveillance improves early identification of high-risk fetuses. Future work should also evaluate how these associations evolve across gestation and whether similar patterns exist in broader obstetric populations beyond fetal cardiology referrals.

### 5.2. Results in the Context of What’s Known

Prior studies have linked thick placenta to adverse outcomes such as fetal growth restriction, preeclampsia, and perinatal morbidity [1,2,3,4,5,6,7,8,9,10,11,12,13,14,15,16]. However, few have explored its relationship with specific fetal conditions using structured classification or weighted severity models. Our findings extend existing knowledge by showing that placental thickness is not only associated with general risk, but also correlates in a graded fashion with cumulative fetal dysfunction, particularly systemic and cardiac soft signs.

The weak association with structural CHDs supports prior work suggesting that many anatomic defects arise early in gestation, while placental adaptations may reflect dynamic responses to ongoing physiologic strain [33,34]. Meanwhile, the stronger correlation with functional cardiac signs and extracardiac soft markers highlights placental thickness as a possible proxy for evolving fetal compromise late in pregnancy.

### 5.3. Clinical Implications

Although placental thickness is not routinely measurable during third-trimester ultrasound, it requires no additional equipment or imaging time [35,36,37,38]. Our findings suggest that a placenta measuring ≥70 mm, though relatively uncommon, may warrant increased diagnostic vigilance. This could include more detailed fetal echocardiography, Doppler assessments, or repeat imaging to monitor for progression.

Importantly, thick placentas appear more predictive of functional and systemic disturbances than structural anomalies. This distinction may help clinicians refine their triage and follow-up protocols. Rather than prompting referral solely for suspected malformations, placental thickness may serve as an early indicator of global fetal dysregulation, especially when combined with other soft markers.

In resource-limited settings, where comprehensive fetal evaluation may not be feasible for all patients, placental thickness could function as a screening adjunct, guiding selective referral or closer surveillance in high-risk pregnancies.

### 5.4. Research Implications

This study introduces a severity-weighted classification system that may be adapted to other areas of prenatal imaging analysis. Future research should validate the proposed thresholds and scoring system in independent cohorts, ideally in a prospective design. Longitudinal studies tracking fetal outcomes into the neonatal period could help determine whether elevated placental thickness predicts measurable postnatal complications.

Moreover, integration of placental metrics into machine learning models or AI-supported diagnostic tools may enable more accurate risk stratification and targeted surveillance. It remains to be seen whether combining placental thickness with fetal biometry, Doppler indices, or biomarker panels could enhance predictive accuracy for perinatal outcomes in high-risk populations.

### 5.5. Strengths and Limitations

One of the key strengths of this study is the large, clinically diverse cohort, drawn from a high-volume fetal cardiology center with standardized imaging protocols. The application of a condition weighting system based on mortality risk adds granularity to the analysis and avoids overrepresenting mild or incidental findings. Additionally, the use of placental thickness as a continuous variable enabled correlation analysis that reflects physiologic trends rather than arbitrary thresholds.

However, the retrospective nature of the study introduces potential bias in both data gathering and diagnosis classification. Placental thickness was not available in approximately half of all fetal echocardiograms performed during the study period, and selection bias may exist if thickness was more likely to be reported in concerning cases. Although we attempted to minimize misclassification by using structured diagnostic categories and consistent mappings, inter-operator variability in ultrasound reporting cannot be excluded. Lastly, this cohort reflects a referred population rather than the general obstetric population, limiting generalizability.

## 6. Conclusions

When the placenta is notably thicker than usual, it seems to go hand in hand with a heavier load of fetal complications overall. Though it’s not enough on its own for a full diagnosis, this measurement provides a straightforward, easy-to-obtain indicator of potential risks during the third trimester—one that might be overlooked more often than it should. Adding placental thickness to standard fetal monitoring routines could help spot functional heart problems and other systemic issues earlier, allowing for better prioritization in at-risk pregnancies. That said, we need more studies across varied populations to really nail down its place in everyday prenatal practice.

## Figures and Tables

**Table 1:** Classification of extracardiac malformations (ECM) by anatomical severity and expected contribution to fetal or neonatal mortality. Includes suggested relative weights for composite outcome modeling [27].

ECM Severity Class	Medical Staff for Newborn	Expected Life Span of Fetus/Neonate	Place of Birth	Treatment Urgency/Type	Rationale (For)	Example ECMs	Suggested Mortality Weight
Heaviest ECMs	Neonatologist, pediatric surgeon, palliative care team, genetics consult	Extremely limited (hours to days), often prenatal demise	Major tertiary center with maximal support	Palliative care or experimental only	Defect is incompatible with life or repair is high-risk	Bilateral renal agenesis, severe lung agenesis, anencephaly, extreme skeletal dysplasias	1.0 (e.g., bilateral renal agenesis, anencephaly, lethal skeletal dysplasia)
Critical ECMs	Neonatal intensivists, pediatric surgeons, subspecialists (urology, GI, neurosurgery)	Survival possible with prompt intervention (days to weeks)	Tertiary care hospital/children’s hospital	Emergency or urgent postnatal surgery/intervention	Defect threatens survival, repair possible	Congenital diaphragmatic hernia, large omphalocele, tracheoesophageal fistula, giant SCT	0.7–0.8 (e.g., CDH, large omphalocele, TE fistula, giant SCT)
Heavy planned ECMs	Neonatologist, pediatric surgical team, relevant subspecialists	Good survival with management (weeks to years)	Major children’s hospital/referral center	Planned surgery early in neonatal period	Repair improves outcome but not immediately required	Moderate omphalocele, bladder exstrophy, renal dysplasia, Hirschsprung’s disease	0.3–0.4 (e.g., moderate omphalocele, bladder exstrophy, Hirschsprung’s)
Not urgently ECMs	General neonatology, pediatric follow-up, subspecialist consultation	Near-normal life expectancy (if isolated)	Local hospital with ability to refer	Elective surgery, possibly delayed	Defect does not threaten life; correction can be delayed	Mild hydronephrosis, cleft lip, small hernias, minor limb anomalies	0.0–0.1 (e.g., mild hydronephrosis, cleft lip, small hernia, limb anomalies)

**Table 2:** CWe categorize congenital heart defects (CHD) by looking at their anatomical complexity, whether surgery is needed, and the level of mortality risk involved. The weights we’ve proposed here highlight their relative importance when it comes to prognosis [25].

CHD Severity Class	Medical Staff for Newborn	Expected Life Span of Fetus/Neonate	Place of Birth	Treatment Urgency/Type	Rationale (For)	Example CHDs	Suggested Mortality Weight
Most Severe CHDs	Prepared neonatal team	Prenatal death likely; high mortality	Specialist hospital (tertiary)	No surgical option; palliative care only	Anomalies incompatible with life or untreatable	HLHS with closed FO, TAPVR with obstruction, Ebstein’s anomaly with cardiomegaly, fetal heart failure- FHF	1.0
Critical CHDs	Obstetrician, neonatologist, pediatric cardiologist, echocardiographer, surgical team	Prenatal period; possible death shortly after birth unless treated immediately	Special delivery room, referral center (e.g., ICZMP Łódź, UMed Warsaw)	Immediate postnatal intervention or surgery	Threatens life shortly after birth without urgent care	HLHS with FO restriction, D-TGA with FO restriction, critical PS or AS, ectopia cordis	0.8–0.9
Severe CHDs	Obstetrician, neonatologist, pediatric cardiologist, planned diagnostics, NICU	Prenatal or early neonatal period; possible survival after intervention	Cardio-obstetric reference center (e.g., ICZMP Łódź, UMed Warsaw, Kraków-Prokocim, Gdańsk-GUM)	Planned early surgery, postnatal stabilization	Treatable with early planning; requires specialized center	D-TGA without FO restriction, HLHS without FO restriction, complex CHDs	0.4–0.6
Planned- Non-Urgent CHDs	Obstetrician, neonatologist, pediatric cardiologist (outpatient follow-up)	Prenatal and neonatal periods; good long-term prognosis with planned treatment	Any maternity hospital	Elective surgery during infancy	Stable CHDs manageable with long-term planning	VSD, AVSD, mild valve abnormalities	0.1–0.2
Non-urgent	Pediatrician/Neonatologist	Usually survives infancy	Any general pediatric center	Observation or delayed surgery	Mild form; rarely needs early intervention	TOF with mild narrowing	0.05

**Table 3:** Classification of fetal cardiac dysfunctions as observed via prenatal echocardiography, stratified by hemodynamic severity and anticipated mortality impact. Includes proposed weightings for risk stratification [26].

Cardiac Dysfunction Severity Class	Medical Staff for Newborn	Expected Life Span	Place of Birth	Treatment Urgency/Type	Rationale (For)	Example Cardiac Dysfunctions	Suggested Mortality Weight
Severe (Life-threatening dysfunctions)	Neonatologist, pediatric cardiologist, NICU team	Shortened without intervention; may lead to fetal or neonatal death	Tertiary care center with cardiology and intensive care	Immediate or early intervention postnatally; prenatal monitoring critical	Cardiac dysfunction may severely compromise circulation or heart function	Fetal heart failure, fetal circulatory failure, hypertrophic cardiomyopathy	0.8–1.0
Moderate (Requires monitoring and possible intervention)	Pediatric cardiologist, neonatologist	Normal if managed appropriately	Specialized center or hospital with cardiology consultation available	Postnatal cardiologic follow-up and intervention if indicated	May affect function or progress if left unmonitored	Valve regurgitations (mitral, tricuspid, pulmonary, aortic) in structurally normal hearts, increased pulmonary resistance	0.3–0.6
Mild or Functional (Benign or transient findings)	General pediatrician, cardiologist if needed	Normal; usually self-resolving or non-progressive	Any hospital with routine pediatric care	No intervention needed, monitor during routine care	Typically benign and not associated with structural heart disease	Echogenic focus (bright spot), chordae tendineae, extrasystoles, false-positive coarctation of aorta, foramen ovale anomalies	0.0–0.2

**Table 4:** Classification of non-cardiac, functional fetal abnormalities (e.g., placental, thoracic, or neurological dysfunctions), with estimated weights representing their prognostic contribution to adverse outcomes [26,27].

Extracardiac Dysfunction Severity Class	Medical Staff for Newborn	Expected Life Span	PLACE OF BIRTH	Treatment Urgency/Type	Rationale (For)	Example Extracardiac Dysfunctions	Suggested Mortality Weight
Severe (Life-threatening or major complications)	Neonatologist, multidisciplinary team (surgeon, geneticist, intensivist)	Shortened without intervention or associated with high morbidity	Tertiary perinatal center with surgical and NICU capabilities	Immediate intervention or intensive postnatal monitoring required	These conditions can severely compromise fetal well-being or signal systemic disease	Hydrops, ascites, pericardial/pleural effusion, meconium peritonitis, IUGR, severe Doppler flow reversal	0.7–1.0
Moderate (Requires monitoring and may need intervention)	Neonatologist, pediatric subspecialist as needed	Likely normal if monitored and treated properly	Hospital with neonatal support and access to pediatric care	Postnatal evaluation and planned treatment if necessary	May indicate underlying issues or cause complications if missed	Polyhydramnios, oligohydramnios, ventriculomegaly, pyelectasis, hepatomegaly, gallbladder enlargement, abnormal MCA flow	0.3–0.6
Mild or Transient (Benign or usually self-limited)	General pediatrician, outpatient follow-up if needed	Normal; often no intervention needed	Any standard maternity hospital	Observation or reassurance, outpatient follow-up if persistent	Typically minor findings that resolve or have limited clinical relevance	Chorionic plexus cysts (CPC), hiccups, nuchal cord, pseudoknot, femur shortening, single umbilical artery	0.0–0.2

## Data Availability

The data that support the findings of this study were derived from the internal fetal echocardiographic database of the Polish Mother’s Memorial Hospital Research Institute. Due to patient privacy and institutional regulations, the raw datasets are not publicly available. De-identified data may be made available from the corresponding author upon reasonable request and with permission from the Polish Mother’s Memorial Hospital Research Institute.

## Glossary of Terms

Placental Thickness (PT)	The maximum perpendicular measurement of the placenta from top to bottom edge on ultrasound, excluding cord insertion. Reported in millimeters (mm).
Condition Classifications	Groupings of fetal diagnoses into four clinically defined domains:
Congenital Heart Defects (CHD)	Structural abnormalities of the fetal heart.
Cardiac Dysfunction (CD)	Functional disturbances of the heart (e.g., valve insufficiency, cardiomegaly, abnormal flow patterns).
Extracardiac Malformations (ECM)	Structural anomalies outside the heart (e.g., CNS, GI, GU, musculoskeletal).
Extracardiac Dysfunction (ED)	Non-structural soft markers of systemic fetal stress (e.g., ventriculomegaly, pyelectasis, abnormal amniotic fluid).
Severity Weight	A numerical score assigned to each diagnosis based on its relative fetal or neonatal mortality risk. Used to reflect the clinical impact of each condition beyond simple presence/absence.
Weighted Score	The sum of severity weights for all diagnoses present in a fetus, calculated separately for each Condition Classification.
Spearman’s ρ (rho)	A non-parametric correlation coefficient used to assess monotonic relationships between variables. Values range from −1 (perfect inverse) to +1 (perfect direct correlation).
Kruskal–Wallis Test	A non-parametric method for comparing more than two independent groups on a continuous or ordinal outcome.
Mann–Whitney U Test	A non-parametric alternative to the *t*-test for comparing two independent groups on a continuous or ordinal variable.
